# A novel styryl diphenylamine derivative reverts the transformed phenotype of human fibrosarcoma HT1080 cells.

**DOI:** 10.1038/bjc.1995.489

**Published:** 1995-11

**Authors:** I. Ohizumi, M. Tanemura, S. Kaihoh

**Affiliations:** Department of Cancer Research, Fuji Gotemba Research Laboratories, Chugai Pharmaceutical Co. Ltd., Shizuoka, Japan.

## Abstract

**Images:**


© 1995 Stockton Press All rights reserved 0007–0920/95 $12.00

### A novel styryl diphenylamine derivative reverts the transformed phenotype of human fibrosarcoma HT1080 cells

I Ohizumi, M Tanemura and S Kaihoh

*Department of Cancer Research, Fuji Gotemba Research Laboratories, Chugai Pharmaceutical Co. Ltd, 135 Komakado 1 Chome, Gotemba-shi, Shizuoka 412, Japan.*

**Summary** Revertant cells, which can be isolated from transformed cells, are flat, non-transformed variants that have contributed to the elucidation of mechanisms involved in cell transformation. We have discovered that a novel styryl diphenylamine derivative converts human fibrosarcoma HT1080 cells into revertant cells. This compound induces flat cell morphology and causes a decrease in proliferative rate. The flat revertant cells not only exhibit a reduction in saturation density at confluence, but also lose the ability to proliferate in soft agar. Furthermore, their tumorigenicity is reduced when injected s.c. into athymic nude mice. The compound alters morphology in three out of seven cancer cell lines and has a potent growth inhibitory effect in six of these lines. In contrast, it has only low levels of cytotoxicity for three normal diploid cell lines. These findings indicate that this styryl diphenylamine derivative has the potential to suppress the malignant phenotype of cancer cells without profound cytotoxicity in non-transformed cells.

**Keywords:** styryl diphenylamine derivative; human fibrosarcoma HT1080; saturation density; tumorigenicity; flat revertant

Revertant cells are flat, non-transformed variants that can be isolated from populations of transformed cells through artificial techniques (Wyke, 1971; Gazdar *et al.*, 1974; Vogel and Pollack, 1974; Varmus *et al.*, 1981). Such isolated revertant cells may be a useful tool for understanding mechanisms of tumorigenic transformation and it is apparent that studies of revertant cells have contributed to the progress of molecular oncology. To date, a variety of compounds which revert the phenotype of oncogene-transfected cells have been reported: azatyrosin (Krzyzosiak *et al.*, 1992; Kyprianou *et al.*, 1992), okadaic acid (Sakai *et al.*, 1989), herbimycin A (Uehara *et al.*, 1989), tyrosine kinase inhibitors (Umezawa *et al.*, 1991), trichostatin A (Sugita *et al.*, 1992*a*) and depudecin (Sugita *et al.*, 1992*b*); most of these have been isolated from micro-organism cultured media.

We have discovered a synthetic low molecular weight compound, a styryl diphenylamine derivative, which induces flat revertants in human fibrosarcoma HT1080 cells derived from naturally occurring tumours. In the initial study we screened derivatives of lobenzarit disodium, an agent used to treat rheumatoid arthritis (Ohsugi *et al.*, 1983), to study the modulation of cytokine induction. We noticed that a few of the derivatives could directly alter the morphology of certain tumour cell lines which expressed a flat revertant-like phenotype. Among these, HT1080 cells were found to be an appropriate candidate in terms of exhibiting definite alterations in cell morphology. Since we were interested in exploring new compounds that have a stronger ability to alter the morphology of HT1080 cells into the flat revertant, we started to synthesise scores of such derivatives. Finally, we found a novel and potent derivative, RX-465, dissimilar to conventional anti-cancer drugs in chemical structure. We predicted that the styryl diphenylamine derivative would be able to reduce the malignant phenotype of tumour cells, because the cell morphology induced by this compound is closely related to that of the flat revertant, showing reduction of mutant N-*ras* gene product p21 ($p21^{N\text{-}ras}$), which participates in the control of this transformed phenotype as shown in HT1080 cells using a suicide technique (Paterson *et al.*, 1987).

In the present paper, we describe how revertant cells induced with this novel styryl diphenylamine derivative are transformed back from human malignant fibrosarcoma HT1080 cells and lose their malignancy in terms of rapid proliferation rate with cellular piling, a high saturation density at confluence, anchorage-independent growth in soft agar and tumorigenicity in nude mice. We also show the effects of this compound on other cancer cell lines and some normal diploid cell lines in terms of growth and morphology.

Correspondence: I Ohizumi
Received 18 October 1994; revised 1 March 1995; accepted 29 May 1995

### Materials and methods

### *Compound*

A styryl diphenylamine derivative, RX-465 (Figure 1), was synthesised in our laboratory. A 10 mM stock solution of the compound was prepared in dimethylsulphoxide (DMSO) and diluted with medium before addition to cells. The final concentration of DMSO in the medium was 0.1%, and had no effect on the morphology or growth of HT1080 cells as well as other cell lines used in this paper.

### *Cell culture*

HT1080 human fibrosarcoma, K-BALB Kirsten murine sarcoma virus-transformed BALB/3T3, L-929 derived from murine connective tissue, T24 human bladder carcinoma, PA-1 human ovarian teratocarcinoma, human diploid fibroblast MRC-5, WI-38 and IMR-90 were purchased from ATCC (Rockville, MD, USA). P388 murine leukaemia and B16 murine melanoma were obtained from the Cancer Chemotherapy Center, Japanese Foundation for Cancer Research (Tokyo, Japan). All tumour cell lines were cultured in Dulbecco's modified Eagle medium (DMEM; Gibco,

$C_{22}H_{19}NO_2$ Mol. wt. 329

**Figure 1** Chemical structure of RX-465.

Grand Island, NY, USA) containing 10% fetal bovine serum (FBS) (Gibco) under 5% carbon dioxide in 100% humidified air at 37°C. Normal diploid cell lines were cultured in Eagle's minimum essential medium (Gibco) containing 10% FBS. The cell lines were tested for and found to be free of mycoplasma contamination with a mycoplasma T.C. rapid detection system (Gen-Probe, San Diego, CA, USA).

*Cell growth assay*

Cells were inoculated into a 24-well plate (Falcon, Lincoln Park, NJ, USA) at $2.5 \times 10^4 - 1 \times 10^5$ cells per well on day 0, and the next day were treated with medium either containing various concentrations of RX-465 or in the absence of RX-465 as a control. The medium was changed on days 3 and 5. At the appropriate time interval, the cells were harvested with 0.05% trypsin-0.53 mM EDTA in Hanks' balanced salt solution (HBSS) (Gibco) and the number of cells was counted with a Coulter Counter ZB1 (Coulter Electronics, Hialeah, FL, USA). $IC_{50}$ value (concentration producing 50% cell growth inhibition) was calculated on the basis of the number of cells treated with or without various concentrations of RX-465 when control cells reached a saturation density.

*Anchorage-independent cell growth assay*

HT1080 cells were grown with or without 1 μM, 2 μM RX-465 for 2 days. The cells were harvested with 0.05% trypsin-0.1% EDTA in phosphate-buffered saline (PBS) and washed with fresh medium several times to remove RX-465. After confirming their ability to exclude trypan blue dye, they were plated at $1 \times 10^3$ cells per well onto a 24-well tissue culture plate in 0.3% agar with DMEM supplemented with 10% FBS. After 12 days the number of colonies per well was counted.

*Tumorigenicity assay*

HT1080 cells were grown with or without 2 μM RX-465 for 48 h, and the cells were harvested with 0.05% trypsin-0.1% EDTA in PBS. These cells, freed from RX-465 by a washing procedure, were checked for viability by trypan blue exclusion. Approximately $1 \times 10^6$ viable cells were inoculated s.c. into athymic (*Nu/Nu*) male mice (CSK Research Park, Tokyo, Japan) aged 6 weeks. Animals were fed *ad libitum* an autoclaved diet and tap water and maintained in sterilised cages. After 17 days, the tumours were excised and weighed.

### Results

*Effects of RX-465 on cell morphology of HT1080 cells*

Human fibrosarcoma HT1080 cells, cultured with RX-465 for 48 h at 2 μM, showed an altered morphology (Figure 2). The parental HT1080 cells displayed a spindle-shaped, refractile and disoriented appearance, grew densely and aggregated upon each other, whereas HT1080 cells treated with RX-465 appeared to be larger than the parental cells, acquired flat morphology indistinguishable from normal epithelium-like cells, and grew as a strict monolayer with little evidence of cellular piling.

*Effects of RX-465 on anchorage-dependent growth of HT1080 cells*

As shown in Figure 3, HT1080 cells treated with RX-465 grew at a much slower rate in a dose-dependent manner, compared with parental cells. The doubling time for HT1080 cells treated with 10 μM RX-465 was approximately 79 h, whereas the parental cells showed a doubling time of 20 h. Moreover, HT1080 cells cultured with RX-465 grew to a lower saturation density in a dose-dependent manner on the 8th day after cell plating. The parental cells reached a satura-

**Figure 2** Phase-contrast photomicrographs of HT1080 human fibrosarcoma cells treated with (**b**) or without (**a**) 2 μM RX-465 for 48 h. Bar = 100 μm.

**Figure 3** A decrease in a growth rate and a saturation density in HT1080 human fibrosarcoma cells by treatment with RX-465. HT1080 cells were plated at $2.5 \times 10^4$ cells per well on a 24-well tissue culture plate on day 0. The next day they were treated with medium containing 1 μM (○), 3 μM (△) or 10 μM (□) RX-465 or vehicle control (●). The medium was changed on days 3 and 5. At the appropriate time interval the number of cells was counted. The data obtained represent the averages of measurements made on three wells. Similar results were obtained in three separate experiments.

tion density of $1.6 \times 10^6$ cells per well in a 24-well plate. In contrast, revertant cells treated with 10 µM RX-465 exhibited a growth density that was 10-fold lower than that of the parental cells. The effects of RX-465 on both growth rate and saturation density was not apparently associated with either acute or delayed cytotoxicity; revertant cells which had reached confluence with 10 µM RX-465 were able to exclude trypan blue. On re-inoculation into a culture plate with culture medium containing RX-465, all of the revertant cells were able to attach and grow at almost the same rate as shown in Figure 3 without any cytotoxicity. Removal of this compound from culture medium resulted in restoration to the original morphology and growth rate during passage 3.

*Effects of RX-465 on anchorage-independent growth of HT1080 cells*

RX-465 has a potent, dose-dependent inhibitory effect on anchorage-independent cell growth. Revertant cells induced by RX-465 over a 2 day period showed hardly any colony formation in soft agar, i.e. at only 20% and 10% of control, at 1 µM and 2 µM respectively (Figure 4). The object of eliminating RX-465 from soft agar was to examine the biological characteristics of HT1080 cells at the point of acquiring the observably flat morphology. The revertant cells in soft agar culture without RX-465 retained the loss of this transformed property as distinguished from those in liquid culture.

*Effects of RX-465 on tumorigenicity of HT1080 cells*

In order to investigate whether the RX-465 revertants lose the ability to produce tumours *in vivo*, we examined the effects of RX-465 on the tumorigenicity of HT1080 cells in athymic mice. After treatment with 2µM RX-465 for 48 h, HT1080 cells, freed from RX-465 by a wash procedure and able to exclude trypan blue, were inoculated s.c. into athymic mice. On the 17th day after inoculation, the revertant cells induced with RX-465 grew at a slower rate than those treated with vehicle control, with an 88% reduction in the average weight of the tumours (Figure 5). With regard to tumour incidence, the cells cultured with vehicle control induced tumour formation in all mice. In contrast, no growth of those revertant cells cultured with RX-465 was observed in four out of the eight athymic mice. In addition, these cells failed to produce tumours in the subsequent 4 month observation period of these four mice. Absence of tumour was confirmed by detailed autopsy of one of the four mice.

**Figure 4** Inhibition of anchorage-independent growth of HT1080 human fibrosarcoma cells by treatment with RX-465. HT1080 cells were grown with or without 1 µM or 2 µM RX-465 for 2 days. The cells were harvested and washed to remove RX-465. The viable cells that could exclude trypan blue dye were plated at $1 \times 10^3$ cells per well into a 24-well tissue culture plate in 0.3% agar with DMEM supplemented with 10% FBS. After 12 days the number of colonies forming 0.1 mm in diameter per well was counted. The data obtained represent the average percentage of the colonies made in two wells. Similar results were obtained in two separate experiments.

*Effects of RX-465 on anchorage-dependent growth and cell morphology in tumour cell lines and normal diploid cell lines*

We examined the effect of RX-465 on other cell lines in terms of cell growth and cell morphology to clarify whether the morphological change is restricted only to human fibrosarcoma HT1080 cells. Table I shows that RX-465 had a potent growth-inhibitory effect against L-929, B16 melanoma, K-BALB, P388 leukaemia, and T24 carcinoma as well as HT1080 fibrosarcoma. However, it had little effect on growth of PA-1 teratocarcinoma, diploid fibroblast MRC-5, WI-38 and IMR-90. In other words, RX-465 had approximately 100-fold specificity to the former lines compared with the latter ones. We also found that RX-465 induced flat cell morphology in L-929 and B16 melanoma as observed in HT1080 cells. In contrast, there were some cell lines such as K-BALB, P388 and T24 whose growth was inhibited by RX-465 without morphological changes.

**Figure 5** Inhibition of tumorigenicity of HT1080 human fibrosarcoma cells by treatment with RX-465. HT 1080 cells which had been treated with or without 2 µM RX-465 for 48 h were harvested and washed so as to be free from RX-465. Approximately $1 \times 10^6$ viable cells that could exclude trypan blue dye were inoculated s.c. into athymic nude mice aged 6 weeks. After 17 days, the formed tumours were taken out and measured for weight. Circles, individual tumour weight; bars, mean ± s.e.; $n = 10$ (control group), $n = 8$ (RX-465-treated group). *Significantly smaller than untreated control group ($P < 0.001$). Similar results were obtained in three separate experiments.

**Table I** Effect of RX-465 on anchorage-dependent growth and cell morphology in tumour cell lines and normal diploid cell lines

| *Species* | *Cell line* | *Ras* | $IC_{50}$ *(µM)*[a] | *Flat cell morphology*[b] |
|---|---|---|---|---|
| Murine | L-929 | H[c], K[c] | 0.6 | Yes |
| | B16 | H[d], K[e] | 1.6 | Yes |
| | K-BALB | K[f] | 0.7 | No |
| | P388 | N[g] | 0.8 | No |
| Human | HT1080 | N | 2.0 | Yes |
| | T24 | H[h] | 0.8 | No |
| | PA-1 | N[i] | >100 | No |
| Human | MRC-5 | | 50 | No |
| | WI-38 | | 80 | No |
| | IMR-90 | | >100 | No |

[a]Concentration producing 50% cell growth inhibition when cells with vehicle control reached a saturation density. [b]Morphology was observed after 72 h treatment of 10 µM RX-465. Ras activation described by [c]Abken *et al.* (1990), [d]Prasad *et al.* (1990), [e]Kris *et al.* (1985), [f]Aaronson & Weaver (1971), [g]Liu *et al.* (1993), [h]Arends *et al.* (1993), [i]Tainsky *et al.* (1988).

### Discussion

The studies described here have shown that a novel styryl diphenylamine derivative, RX-465, reduces the malignant phenotype of human fibrosarcoma HT1080 cells, including features such as rapid rates of proliferation accompanied by cellular piling, high saturation density at confluence, anchorage-independent growth in soft agar and tumorigenicity in nude mice. The revertant cells treated with RX-465 displayed a slower growth rate compared with cells treated with control vehicle, accompanied by morphological alterations of a flattened appearance which closely resembled revertants obtained by Paterson *et al.* (1987) as selected from HT1080 cells by a suicide technique. It is apparent that the slower growth rate in HT1080 cells treated with RX-465 could not be attributed to anomalous factors such as either acute or delayed cytotoxicity because the reproducibility in their growth rate was observed when the revertant cells were reinoculated after achieving confluence. As a consequence of the simple reductions in mutant $p21^{N\text{-}ras}$, as observed by Paterson *et al.* (1987), similar morphological changes in HT1080 cells treated with RX-465 might indicate the possibility of a role for this compound in counteracting neoplastic transformation. Moreover, treatment of HT1080 cells with RX-465 resulted in a lower saturation density and inhibition of anchorage-independent growth in soft agar, both of which characteristics have been associated with reduction of tumour formation in mice, the most critical phenotypic measure of cell transformation. (Aaronson and Todaro, 1968; Freedman *et al.*, 1974). In terms of the relationship between the capability of cell transformation *in vitro* and *in vivo*, there are some exceptions. Not all revertant cells which show a decrease in saturation density and a complete loss of capacity for anchorage-independent growth, also show a reduction of tumorigenicity *in vivo* because of phenotypic instability (Haynes and Downing, 1988). In this context the reduction of the tumorigenicity observed with HT1080 cells treated with RX-465 can be understood to demonstrate that the revertant cells have a stable phenotype which can contribute to the reduction of proliferation *in vivo*. Instability of the revertant phenotype after elimination of RX-465 was only observed during passage 3 in monolayer culture. This is perhaps because certain unknown key factors that are epigenetic components which influence growth regulation are abolished in continuous culture. Also, the frequency of reversion was 96% (1569 out of 1635 cells selected at random) after 7 days' treatment with 10 $\mu$M RX-465 in HT1080 cells, which suggests that a small number of the cells that can resist RX-465 treatment were able to proliferate and dominate culture characteristics after passage 3. Some of the characteristics of malignant tumour cells are cell density-independent proliferation, anchorage-independent growth and tumorigenicity in nude mice. A styryl diphenylamine derivative able to diminish these transforming activities could help to elucidate the molecular basis of cell transformation in malignant tumours.

The mechanism by which this styryl diphenylamine derivative reverts the transformed phenotype of human fibrosarcoma HT1080 cells is still unknown. In our initial studies, we speculated on the basis of the study performed by Paterson *et al.* (1987) that the derivative might reduce cellular concentration of mutant $p21^{N\text{-}ras}$. However, we found that the alteration to flat cell morphology was observed in three out of seven cancer cell lines which exhibit *ras* activation, suggesting that RX-465 may not necessarily revert the phenotype of every tumour cell that contains activated *ras* oncogenes. Therefore, we speculate that the cellular reversion is rarely attributed to a depletion of *ras* function, although it is apparent that this phenomenon is not restricted only to HT1080 fibrosarcoma cells. Interestingly, this compound exerted strong growth inhibitory activity in six out of seven cancer cell lines and had little cytotoxicity in three human normal diploid fibroblast lines. Taken together, our results suggest that RX-465 may have specificity for growth inhibition of tumour cells, including some morphological effects. Next we investigated whether this reversion on the transformed phenotype is associated with tyrosine protein kinase (TPK) activity since RX-465 is similar in chemical structure to TPK inhibitors such as erbstatin, classified with 'styryl-based' inhibitors (Smyth *et al.*, 1993). However, on direct assay of TPK activity we found no evidence for a relationship between signal transductions induced by TPK and the reversion by RX-465 (unpublished data). As a consequence, we speculate that RX-465 may play an important role in the counteraction of common cellular events, independent of *ras* functions among L-929, B16 melanoma and HT1080 fibrosarcoma cells.

In conclusion, we have discovered that a styryl diphenylamine derivative, RX-465, reverts the transformed phenotype of human fibrosarcoma cell line HT1080 at low micromolar concentrations. RX-465 induces a flat cell morphology and a reduction in the proliferative rate. Moreover, the revertant cells exhibit a low saturation density at confluence, lose the ability to proliferate in soft agar and were for the most part unable to induce tumour formation in athymic mice. RX-465 appears to exert no cytotoxic effects on untransformed cells. It is tempting to speculate that RX-465 may be important in cancer chemotherapy.

### Abbreviations

HBSS, Hanks' balanced salt solution; PBS, $Ca^{2+}$- and $Mg^{2+}$-free phosphate-buffered saline; s.c., subcutaneous; $IC_{50}$, concentration producing 50% cell growth inhibition; Hepes, 4-(2-hydroxyethyl)-1-piperazineethanesulphonic acid.

### Acknowledgements

We are grateful to Drs Kevin Boru and Peter Kowalski-Saunders for preparation of the manuscript.

### References

AARONSON SA AND WEAVER CA. (1971). Characterization of murine sarcoma virus (Kirsten) transformation of mouse and human cells. *J. Gen. Virol.*, **13**, 245–252.

AARONSON SA AND TODARO GJ. (1968). Basis for the acquisition of malignant potential by mouse cells cultivated in vitro. *Science*, **162**, 1024–1026.

ABKEN H, BUTZLER C AND WILLECKE K. (1990). Altered expression of proto-oncogenes in human lymphoid cells immortalized by transfection with extrachromosomal DNA of mouse L cells. *Anticancer Res.*, **10**, 73–80.

ARENDS MJ, MCGREGOR AH, TOFT NJ, BROWN EJ AND WYLLIE AH. (1993). Susceptibility to apoptosis is differentially regulated by c-myc and mutated Ha-ras oncogenes and is associated with endonuclease availability. *Br. J. Cancer.*, **68**, 1127–1133.

FREEDMAN VH AND SHIN S. (1974). Cellular tumorigenicity in nude mice: correlation with cell growth in semi-solid medium. *Cell*, **3**, 355–359.

GAZDAR AF, STULL HB, CHOPRA HC AND IKAWA Y. (1974). Properties of flat variants of murine sarcoma virus transformed non-producer cells isolated after high-temperature passage. *Int. J. Cancer*, **13**, 219–226.

HAYNES JR AND DOWNING JR. (1988). A recessive cellular mutation in v-*fes*-transformed mink cells restores contact inhibition and anchorage-dependent growth. *Mol. Cell Biol.*, **8**, 2419–2427.

KRIS RM, AVIVI A, BAR-ELI M, ALON Y, CARMI P, SCHLESSINGER J AND RAZ A. (1985). Expression of ki-*ras* oncogene in tumor cell variants exhibiting different metastatic capabilities. *Int. J. Cancer*, **35**, 227–230.

KRZYZOSIAK WJ, SHINDO-OKADA N, TESHIMA H, NAKAJIMA K AND NISHIMURA S. (1992). Isolation of genes specifically expressed in flat revertant cells derived from activated *ras*-transformed NIH3T3 cells by treatment with azatyrosine. *Pro. Natl Acad. Sci. USA*, **89**, 4879–4883.

KYPRIANOU N AND TAYLOR-PAPADIMITRIOU J. (1992). Isolation of azatyrosine-induced revertants from *ras*-transformed human mammary epithelial cells. *Oncogene*, **7**, 57–63.

LIU YB. (1993). Oncogene expression in adriamycin and platinum resistant cell lines. *Chung. Hua. I Hsueh. Tsa Chih.*, **73**, 552–554.

OHSUGI Y, NAKANO T AND HATA S. (1983). A novel antiarthritic agent, CCA (lobenzarit disodium), and the role of thymus-derived lymphocytes in the inhibition of rat adjuvant arthritis. *Immunopharmacology*, **6**, 15–21.

PATERSON H, REEVES BR, HALL A, FURTH M, BOS J, JONES P AND MARSHALL C. (1987). Activated N-*ras* controls the transformed phenotype of HT1080 human fibrosarcoma cells. *Cell*, **51**, 803–812.

PRASAD KN, COHRS RJ AND SHARMA OK. (1990). Decreased expressions of c-myc and H-ras oncogenes in vitamin E succinate induced morphologically differentiated murine B-16 melanoma cells in culture. *Biochem. Cell Biol.*, **68**, 1250–1255.

SAKAI R, IKEDA I, KITANI H, FUJIKI H, TAKAKU F, RAPP U, SUGIMURA T AND NAGAO M. (1989). Flat reversion by okadaic acid of *raf* and *ret*-II transformants. *Pro. Natl Acad. Sci. USA*, **86**, 9946–9950.

SMYTH MS, STEFANOVA I, HORAK ID AND BURKE JR TR. (1993). Hydroxylated 2-(5′-Salicyl) naphthalenes as protein–tyrosine kinase inhibitors. *J. Med. Chem.*, **36**, 3015–3020.

SUGITA K, KOIZUMI K AND YOSHIDA H. (1992*a*). Morphological reversion of *sis*-transformed NIH3T3 cells by trichostatin A. *Cancer Res.*, **52**, 168–172.

SUGITA K, YOSHIDA H, MATSUMOTO M AND MATSUTANI S. (1992*b*). A novel compound, depudecin, induces production of transformation to the flat phenotype of NIH3T3 cells transformed by *ras*-oncogene. *Biochem. Biophys. Res. Comm.*, **182**, 379–387.

TAINSKY MA, KRIZMAN DB, CHIAO PJ, YIM SO AND GIOVANELLA BC. (1988). PA-1, a human cell model for multistage carcinogenesis: oncogenes and other factors. *Anticancer Res.*, **8**, 899–913.

UEHARA Y, MURAKAMI Y, SUGIMOTO Y AND MIZUNO S. (1989). Mechanism of reversion of Rous sarcoma virus transformation by herbimycin A. *Cancer Res.*, **49**, 780–785.

UMEZAWA K, TANAKA K, HORI T, ABE S, SEKIZAWA R AND IMOTO M. (1991). Induction of morphological change by tyrosine kinase inhibitors in Rous sarcoma virus-transformed rat kidney cells. *FEBS Lett.*, **279**, 132–136.

VARMUS HE, QUINTRELL N AND ORTIZ S. (1981). Retroviruses as mutagenes. *Cell*, **25**, 23–36.

VOGEL A AND POLLACK R. (1974). Methods for obtaining revertants of transformed cells. *Methods Cell Biol.*, **8**, 75–92.

WYKE JA. (1971). Method of isolating cells incapable of multiplication in suspension culture. *Exp. Cell Res.*, **66**, 203–208.


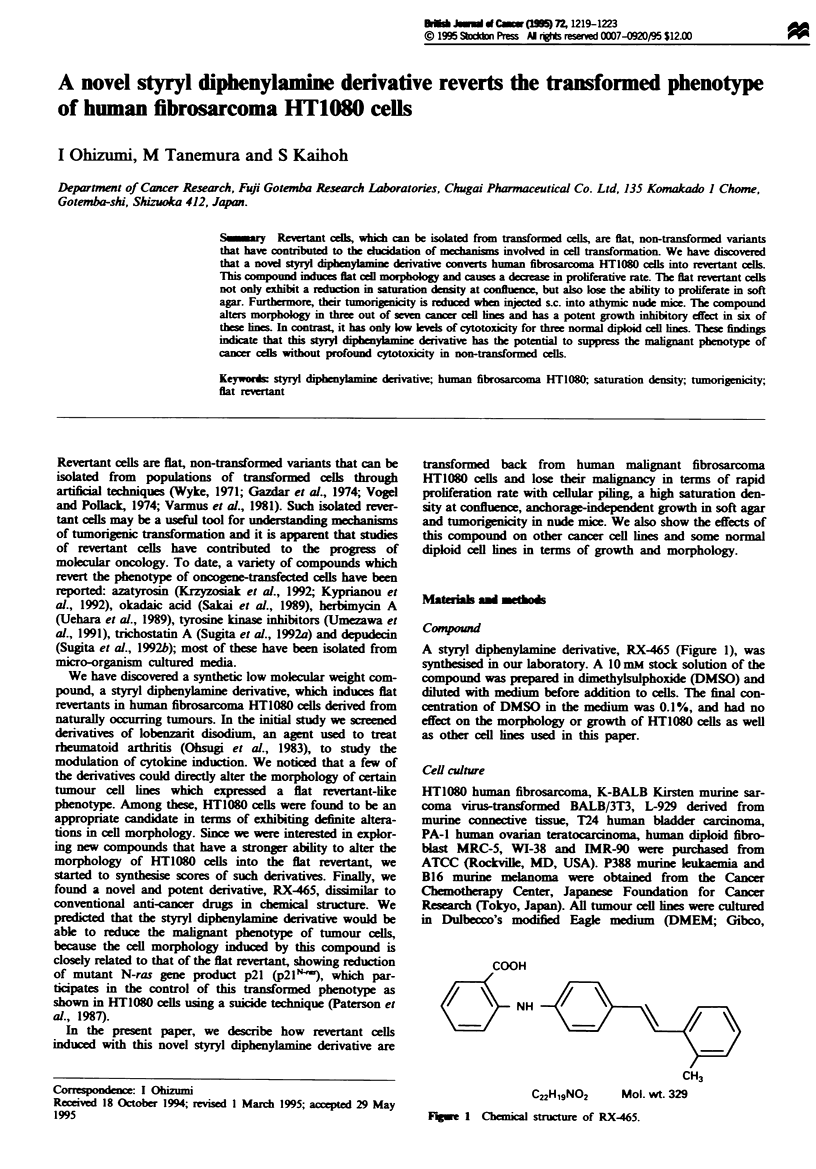

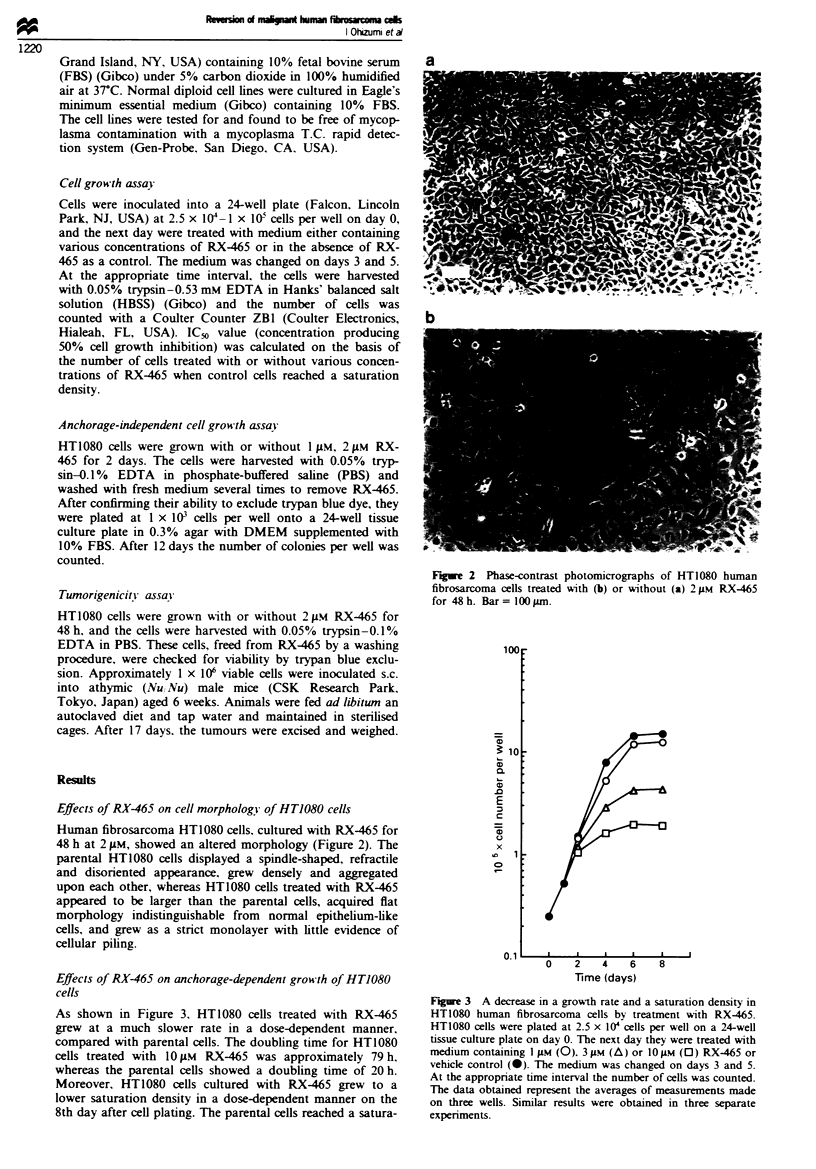

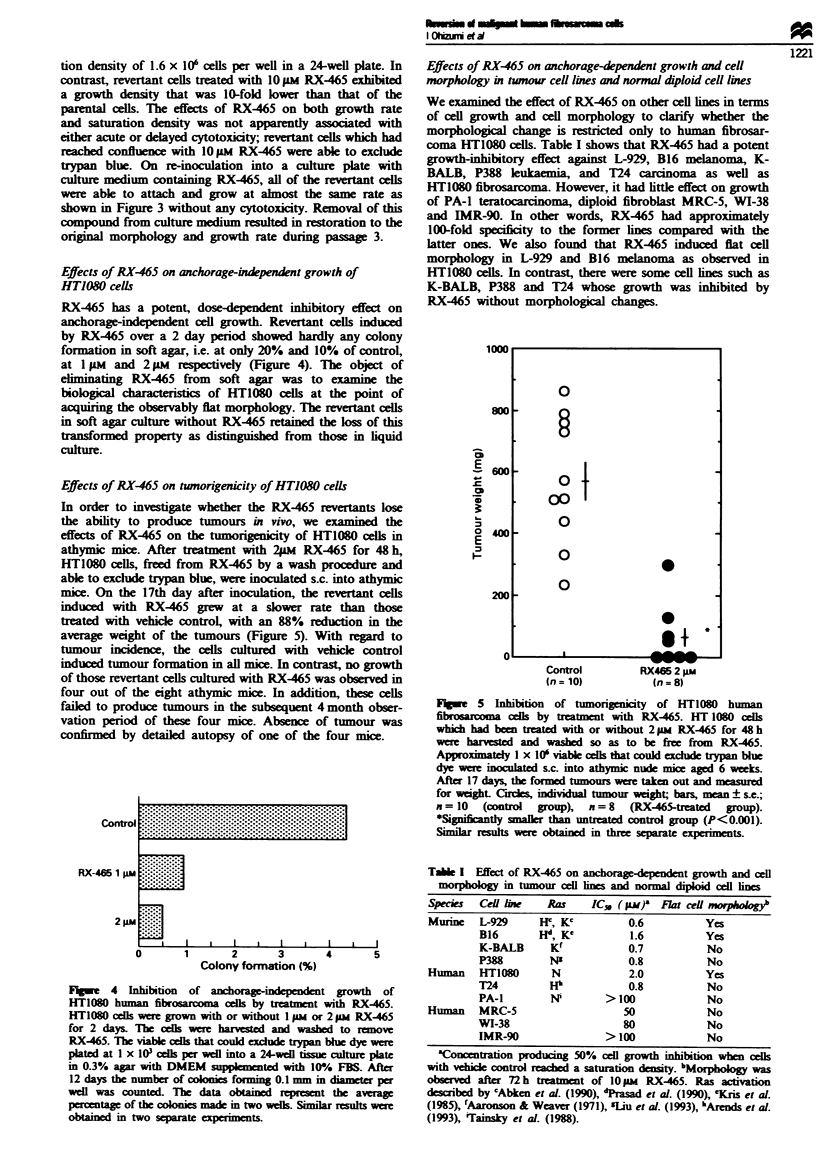

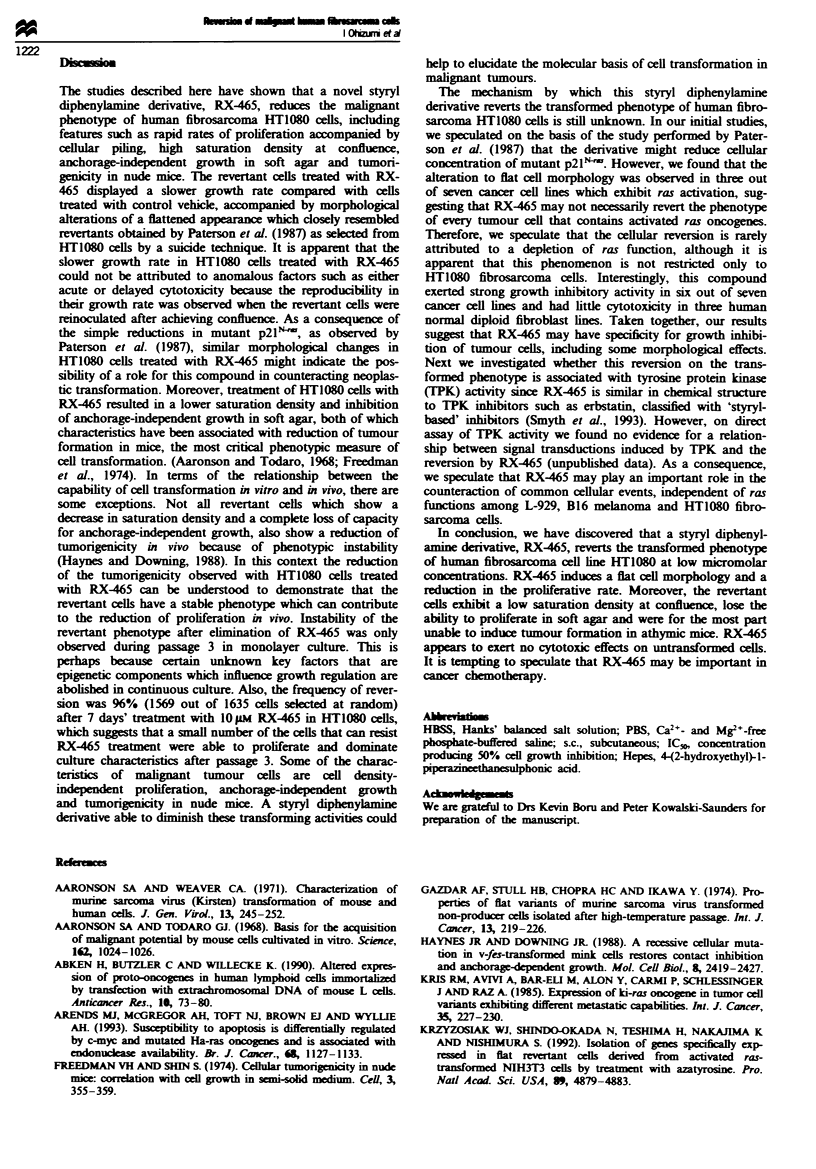

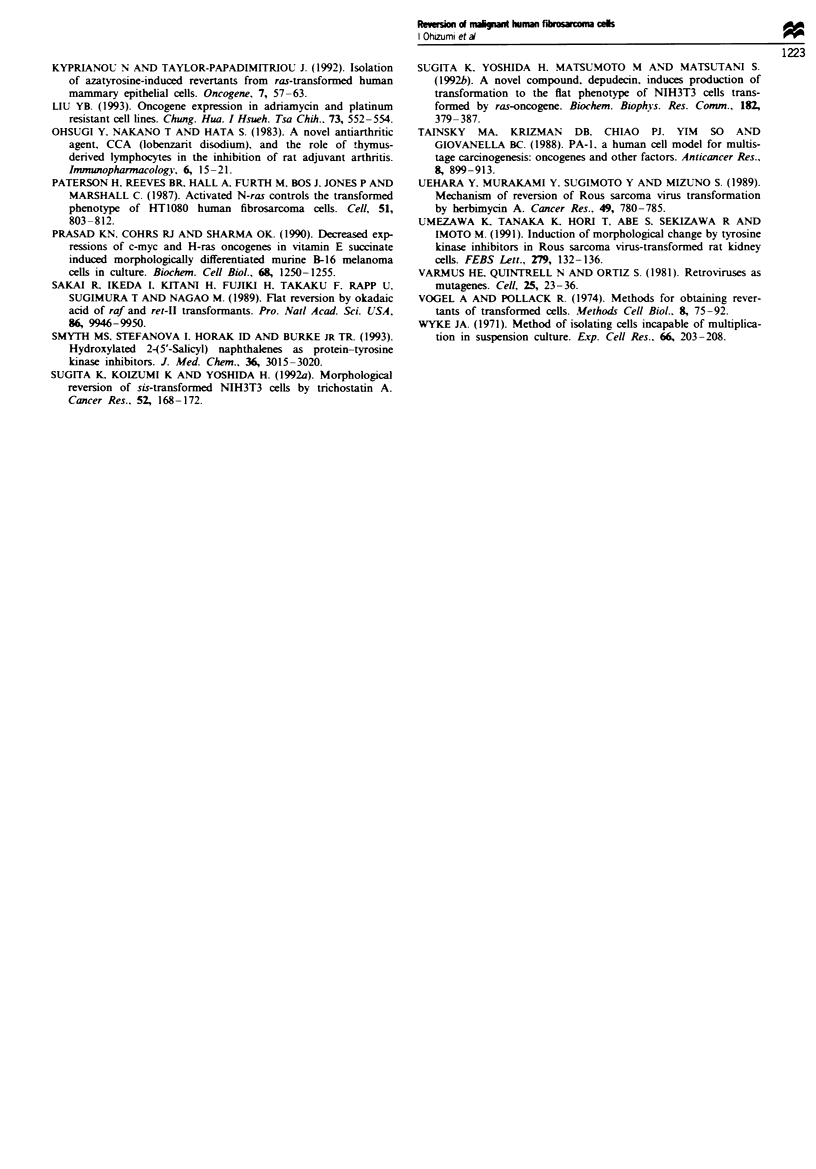


## References

[OCR_00575] Aaronson S. A., Todaro G. J. (1968). Basis for the acquisition of malignant potential by mouse cells cultivated in vitro.. Science.

[OCR_00568] Aaronson S. A., Weaver C. A. (1971). Characterization of murine sarcoma virus (Kirsten) transformation of mouse and human cells.. J Gen Virol.

[OCR_00580] Abken H., Bützler C., Willecke K. (1990). Altered expression of proto-oncogenes in human lymphoid cells immortalized by transfection with extrachromosomal DNA of mouse L cells.. Anticancer Res.

[OCR_00587] Arends M. J., McGregor A. H., Toft N. J., Brown E. J., Wyllie A. H. (1993). Susceptibility to apoptosis is differentially regulated by c-myc and mutated Ha-ras oncogenes and is associated with endonuclease availability.. Br J Cancer.

[OCR_00592] Freedman V. H., Shin S. I. (1974). Cellular tumorigenicity in nude mice: correlation with cell growth in semi-solid medium.. Cell.

[OCR_00595] Gazdar A. F., Stull H. B., Chopra H. C., Ikawa Y. (1974). Properties of flat variants of murine sarcoma virus transformed non-producer cells isolated after high-temperature passage.. Int J Cancer.

[OCR_00603] Haynes J. R., Downing J. R. (1988). A recessive cellular mutation in v-fes-transformed mink cells restores contact inhibition and anchorage-dependent growth.. Mol Cell Biol.

[OCR_00605] Kris R. M., Avivi A., Bar-Eli M., Alon Y., Carmi P., Schlessinger J., Raz A. (1985). Expression of ki-ras oncogene in tumor cell variants exhibiting different metastatic capabilities.. Int J Cancer.

[OCR_00614] Krzyzosiak W. J., Shindo-Okada N., Teshima H., Nakajima K., Nishimura S. (1992). Isolation of genes specifically expressed in flat revertant cells derived from activated ras-transformed NIH 3T3 cells by treatment with azatyrosine.. Proc Natl Acad Sci U S A.

[OCR_00623] Kyprianou N., Taylor-Papadimitriou J. (1992). Isolation of azatyrosine-induced revertants from ras-transformed human mammary epithelial cells.. Oncogene.

[OCR_00630] Liu Y. B. (1993). [Oncogene expression in adriamycin and platinum resistant cell lines].. Zhonghua Yi Xue Za Zhi.

[OCR_00633] Ohsugi Y., Nakano T., Hata S. (1983). A novel antiarthritic agent, CCA (lobenzarit disodium), and the role of thymus-derived lymphocytes in the inhibition of rat adjuvant arthritis.. Immunopharmacology.

[OCR_00639] Paterson H., Reeves B., Brown R., Hall A., Furth M., Bos J., Jones P., Marshall C. (1987). Activated N-ras controls the transformed phenotype of HT1080 human fibrosarcoma cells.. Cell.

[OCR_00645] Prasad K. N., Cohrs R. J., Sharma O. K. (1990). Decreased expressions of c-myc and H-ras oncogenes in vitamin E succinate induced morphologically differentiated murine B-16 melanoma cells in culture.. Biochem Cell Biol.

[OCR_00649] Sakai R., Ikeda I., Kitani H., Fujiki H., Takaku F., Rapp U., Sugimura T., Nagao M. (1989). Flat reversion by okadaic acid of raf and ret-II transformants.. Proc Natl Acad Sci U S A.

[OCR_00657] Smyth M. S., Stefanova I., Horak I. D., Burke T. R. (1993). Hydroxylated 2-(5'-salicyl)naphthalenes as protein-tyrosine kinase inhibitors.. J Med Chem.

[OCR_00662] Sugita K., Koizumi K., Yoshida H. (1992). Morphological reversion of sis-transformed NIH3T3 cells by trichostatin A.. Cancer Res.

[OCR_00667] Sugita K., Yoshida H., Matsumoto M., Matsutani S. (1992). A novel compound, depudecin, induces production of transformation to the flat phenotype of NIH3T3 cells transformed by ras-oncogene.. Biochem Biophys Res Commun.

[OCR_00675] Tainsky M. A., Krizman D. B., Chiao P. J., Yim S. O., Giovanella B. C. (1988). PA-1, a human cell model for multistage carcinogenesis: oncogenes and other factors.. Anticancer Res.

[OCR_00680] Uehara Y., Murakami Y., Sugimoto Y., Mizuno S. (1989). Mechanism of reversion of Rous sarcoma virus transformation by herbimycin A: reduction of total phosphotyrosine levels due to reduced kinase activity and increased turnover of p60v-src1.. Cancer Res.

[OCR_00683] Umezawa K., Tanaka K., Hori T., Abe S., Sekizawa R., Imoto M. (1991). Induction of morphological change by tyrosine kinase inhibitors in Rous sarcoma virus-transformed rat kidney cells.. FEBS Lett.

[OCR_00689] Varmus H. E., Quintrell N., Ortiz S. (1981). Retroviruses as mutagens: insertion and excision of a nontransforming provirus alter expression of a resident transforming provirus.. Cell.

[OCR_00693] Vogel A., Pollack R. (1974). Methods for obtaining revertants of transformed cells.. Methods Cell Biol.

[OCR_00697] Wyke J. (1971). A method of isolating cells incapable of multiplication in suspension culture.. Exp Cell Res.

